# Hemorrhagic Cholecystitis in an Elderly Patient With Fall Syndrome: A Case Report

**DOI:** 10.7759/cureus.114039

**Published:** 2026-08-05

**Authors:** Arturo A Flores Morales, Abelardo Flores Morales, Gil E Belloso Mercado, Rafael Fleites Perez

**Affiliations:** 1 General Surgery, Hospital Civil de Tepic Dr. Antonio Gonzalez Guevara, Tepic, MEX; 2 Cardiology, Centro Médico Nacional Siglo XXI, Mexico City, MEX

**Keywords:** acalculous cholecystitis, elderly, emergency cholecystectomy, fall syndrome, geriatric surgery, hemorrhagic cholecystitis

## Abstract

Hemorrhagic cholecystitis is a rare, potentially fatal complication of acute cholecystitis that develops with or without gallstones. It tends to be diagnosed late because its clinical presentation overlaps with that of other acute abdominal conditions, and suspicion is often low, as it is first a ruling-out diagnosis due to its low incidence. We present the case of an 81-year-old man with epilepsy, heavy tobacco use, and alcohol consumption, who ambulated with crutches due to a previous left ankle fracture. He was admitted following a ground-level fall caused by a tonic-clonic seizure, sustaining a cranio-parietal contusion and direct trauma to the right hemithorax and flank. He was managed for moderate traumatic brain injury with an initial Glasgow Coma Scale score of 15. On the fifth intrahospital day, he developed moderate to severe, constant right-sided abdominal pain with no apparent reason. Laboratory findings showed elevated liver enzymes, amylase, and lipase. Abdominal ultrasound identified a distended gallbladder (GB) containing 200 mL of hyperdense material without acoustic shadowing and free intraperitoneal fluid consistent with hemoperitoneum. Emergency traditional cholecystectomy revealed approximately 100 mL of hemoperitoneum, a fundal hematoma with adherent omental plastron, and a tensely distended intrahepatic GB measuring 8 × 6 × 5 cm with 5-mm walls and a well-organized intraluminal clot. No gallstones were found, confirming acalculous etiology. The patient recovered without vasopressor support and was discharged on the second day after surgery. This case is, to our knowledge, the first indexed report linking acalculous hemorrhagic cholecystitis to geriatric fall syndrome. A systematic search of PubMed, OpenAIRE, and Google Scholar yielded no previously published cases with this clinical association. Early imaging and prompt surgical intervention were the determining factors in this patient's favorable outcome.

## Introduction

Hemorrhagic cholecystitis is a rare, acute, and potentially life-threatening complication of cholecystitis that occurs with or without the presence of gallstones. The underlying pathophysiological cascade begins when inflammatory mediators, ischemia, or direct mechanical injury compromise the integrity of the gallbladder (GB) mucosa, producing ulceration and progressive necrosis of the wall. In the setting of acalculous disease, bile stasis plays a central role: in the absence of a cholecystokinin stimulus - as occurs during prolonged fasting or critical illness - concentrated bile remains stagnant within the lumen, and endotoxins generated by this process have been shown to cause mucosal necrosis, hemorrhage, fibrin deposition, and extensive mucosal loss, consistent with an acute ischemic insult. As this process advances, intraluminal blood accumulates, the GB distends under pressure, and eventually ruptures, releasing hemorrhagic content into the peritoneal cavity. Pathological studies characterize acalculous cholecystitis as a manifestation of systemic disease rather than a local GB disorder, which distinguishes it fundamentally from the calculous form and helps explain why it tends to be diagnosed late and to carry higher mortality. Perforation occurs most often at the fundus, which has the poorest vascular supply and is therefore the most vulnerable to transmural ischemia under tension [1].

Two etiological categories are recognized, and understanding the distinction between them is essential to appreciating why the present case is unusual. The calculous form is more common, occurring in 3 to 10% of calculous cholecystitis cases and typically driven by gallstone-related mucosal injury and obstruction of the cystic duct. The acalculous form is considerably rarer, accounting for 5 to 10% of all cholecystitis cases, and its trigger is not mechanical obstruction but rather one of several systemic or local insults that impair GB perfusion or mucosal defense [2,3]. Recognized precipitants include dehydration, prolonged parenteral nutrition, end-stage renal disease, metabolic and cardiovascular diseases, severe burns, trauma, neoplasia, cardiopulmonary resuscitation, mechanical ventilation, post-transplantation states, and systemic bacterial or viral infections. Additional documented risk factors include anticoagulant therapy, coagulopathies, cirrhosis, vasculitides, and chronic renal impairment. What these conditions share is a common downstream pathway: reduced GB perfusion, mucosal ischemia, loss of the mucosal barrier, and secondary hemorrhage into the lumen. In both categories, diagnosis is frequently delayed because the clinical picture overlaps with that of other acute abdominal conditions - biliary colic, hepatic trauma, hemobilia, or perforated ulcer - and suspicion is often low, particularly in patients presenting with competing diagnoses [4].

The traumatic mechanism of acalculous hemorrhagic cholecystitis deserves specific attention because it is the one most directly relevant to this case. GB contusion after blunt abdominal trauma is a rare event that presents with significant diagnostic challenges, and there is no clear evidence base supporting conservative versus surgical management of GB contusion injuries when they present in isolation. In patients with right upper quadrant pain after blunt trauma and no cholelithiasis, hemorrhagic acalculous cholecystitis should be considered in the differential diagnosis, as imaging findings can be misleading and ultrasound or CT may not reliably differentiate gallstones from intraluminal blood clots. Critically, the onset of symptoms can be delayed by several days after the inciting trauma, as the intraluminal hematoma accumulates gradually before producing obstruction or rupture. Cases of delayed cholecystitis caused by blunt traumatic GB hemorrhage have been described where initial CT imaging was entirely normal, with the diagnosis established only after symptoms worsened and advanced biliary imaging was obtained. This delayed-presentation pattern is particularly relevant in elderly patients sustaining right-flank trauma in the context of a fall, where the traumatic mechanism may be overlooked or attributed to other competing injuries [5]. 

The case described here falls into the acalculous, traumatic category. The patient sustained direct trauma to the right hemithorax and flank during a fall [5,6]. His broader clinical profile meets the criteria for geriatric fall syndrome: an 81-year-old man with a Downton Fall Risk Index (DFRI) of three or more points. The DFRI was originally developed and validated as an inpatient fall risk stratification tool, with a score of two or more indicating high fall risk and an odds ratio of 2.31 for predicting falls during hospitalization [7,8]. Longitudinal data have since demonstrated that a DFRI of three or more is independently associated with fall-related injury after discharge, with an incidence rate ratio of 1.94, confirming that this score captures a clinically meaningful phenotype of recurrent, high-risk falling [9]. What has not been described is the downstream surgical consequence of that falling pattern when right-flank abdominal impact is sustained: the DFRI has not previously been linked to hemorrhagic cholecystitis as a precipitating mechanism [4]. Isolated case reports of hemorrhagic cholecystitis from calculous causes exist in the Mexican literature, and a small number of reports describe the acalculous, traumatic form following direct blunt abdominal impact in younger patients, but an acalculous presentation triggered by fall-related flank trauma in the geriatric population, with high fall risk documented by a validated scoring tool, has not been reported in the indexed literature, making this case presentation of relevance for documentation for its sheer rarity and to take in consideration as a uncommon but present differential diagnosis [10].

This case was previously presented as a research poster at the International Congress organized by the Asociación Mexicana de Cirugía General (AMCG) 2025 Annual Meeting.

## Case presentation

An 81-year-old man with a diagnosis of epilepsy, heavy tobacco use (pack-index: 40), and alcohol consumption presented to the emergency department after an own-height fall. He used crutches for ambulation due to a previous left ankle fracture. His DFRI score was three or more points [8], reflecting high-grade gait alterations, a history of a previous fall requiring hospital admission, and sensory impairment, criteria consistent with geriatric fall syndrome.

The fall was apparently triggered by a tonic-clonic seizure. In addition to a cranio-parietal contusion, the patient sustained blunt trauma to the right hemithorax and right flank. He was admitted and managed conservatively for moderate traumatic brain injury, with a Glasgow Coma Scale score of 15 throughout. His neurological course was uneventful over the first four days.

On the fifth day, he developed moderate to severe, constant pain in the right upper quadrant and right flank. Serum laboratory findings are summarized in Table 1. Values of note included hemoglobin of 11.30 g/dL, hematocrit of 33.90%, leukocyte count of 9.90 x 10³/µL with 89.20% neutrophils, aspartate aminotransferase (AST) activity of 70.3 U/L, alanine aminotransferase (ALT) activity of 49.2 U/L, alkaline phosphatase activity of 146.5 U/L, gamma-glutamyltransferase (GGT) activity of 224.7 U/L, amylase activity of 241 U/L, lipase activity of 202.7 U/L, total bilirubin of 1.01 mg/dL, albumin of 2.17 g/dL, international normalized ratio (INR) of 1.16, and partial thromboplastin time (PTT) of 33.4 s.

**Table 1:** Blood work Findings from the patient's blood work when general surgery made the first assessment on the fifth day of hospital stay. BUN: blood urea nitrogen; AST: aspartate aminotransferase; ALT: alanine aminotransferase; GGT: gamma-glutamyltransferase; INR: international normalized ratio; PTT: partial thromboplastin time

Parameter	Patient value	Reference range
Hemoglobin (g/dL)	11.30	13.5-17.5
Hematocrit (%)	33.90	41-53
Platelets (x10³/µL)	154	150-400
Leukocytes (x10³/µL)	9.90	4.5-11.0
Neutrophils (%)	89.20	50-70
Lymphocytes (%)	6.60	20-40
Glucose (mg/dL)	123.6	70-100
Urea (mg/dL)	68	15-45
BUN (mg/dL)	31.8	7-20
Creatinine (mg/dL)	0.66	0.7-1.3
Sodium (mEq/L)	143	136-145
Potassium (mEq/L)	3.25	3.5-5.0
Chloride (mEq/L)	106.1	98-106
Calcium (mg/dL)	8.07	8.5-10.5
Phosphorus (mg/dL)	5.28	2.5-4.5
Magnesium (mEq/L)	2.46	1.8-2.6
Albumin (g/dL)	2.17	3.5-5.0
Total protein (g/dL)	5.18	6.0-8.3
AST (U/L)	70.3	<40
ALT (U/L)	49.2	<56
Alkaline phosphatase (U/L)	146.5	44-147
GGT (U/L)	224.7	<61
Amylase (U/L)	241	<100
Lipase (U/L)	202.7	<60
Direct bilirubin (mg/dL)	0.50	<0.3
Indirect bilirubin (mg/dL)	0.51	<0.8
Total bilirubin (mg/dL)	1.01	<1.2
Prothrombin time (s)	12.9	11-13.5
INR	1.16	0.8-1.2
PTT (s)	33.4	25-35

**Figure 1:**
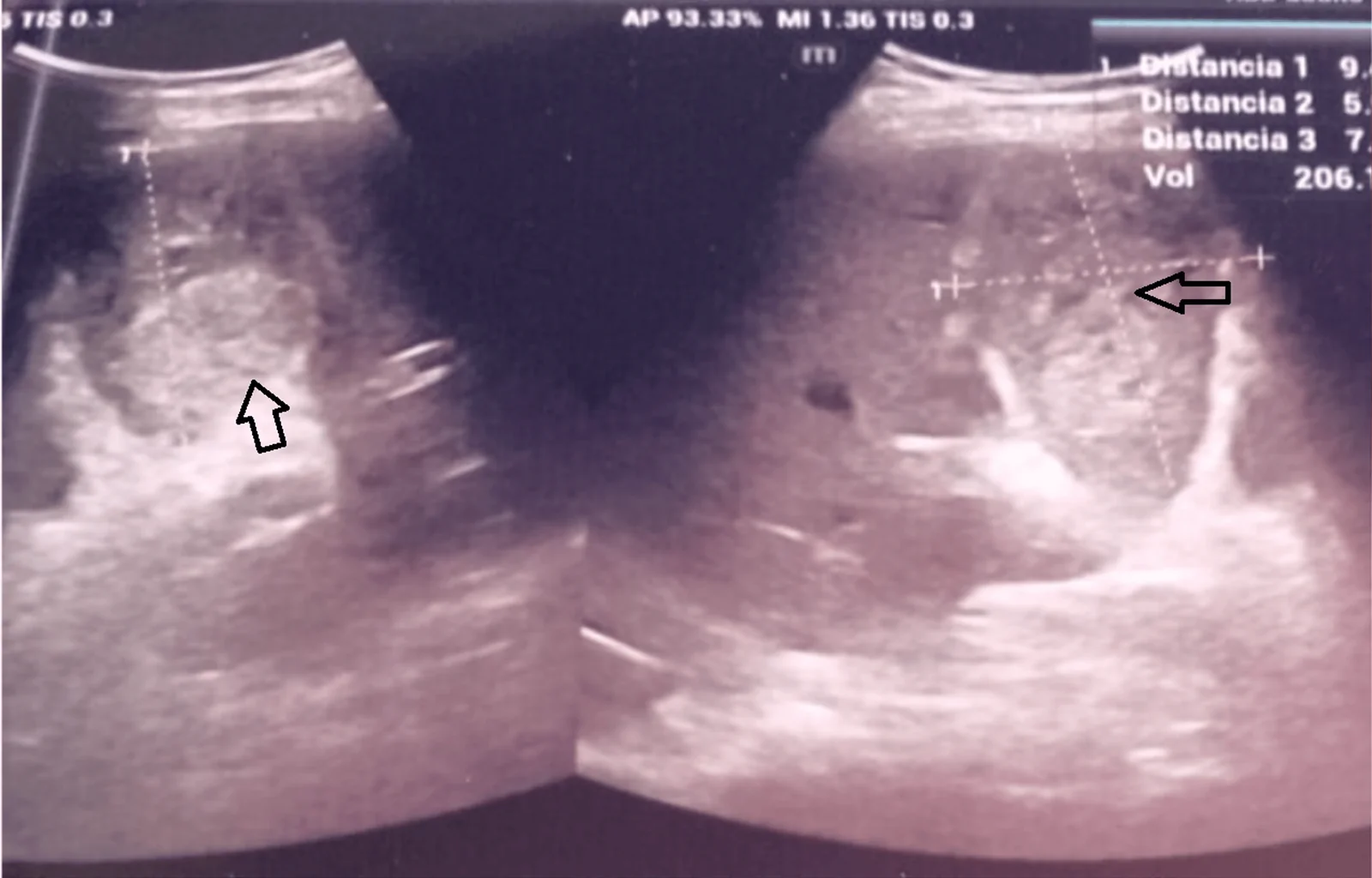
Abdominal ultrasound shows a distended GB with echogenic intraluminal content without acoustic shadowing (arrowheads), consistent with hemorrhagic material. Free intraperitoneal fluid is visible in the hepatorenal space, consistent with hemoperitoneum. The two images are the same ultrasound, with two sides or points of view, printed in the same sheet/film. GB: gallbladder

Abdominal ultrasound (Figure 1) demonstrated a distended GB containing approximately 200 mL of hyperdense material without acoustic shadowing and free intraperitoneal fluid consistent with hemoperitoneum. At the beginning, while being in the emergency department, there was no indication of an acute abdominal etiology that needed surgical management. After the acute pain was established, the suspicion was that the patient could develop hemoperitoneum, diaphragmatic hernia, or even a contained liver rupture. Nevertheless, vital signs were stable, and blood work findings did not indicate a major or catastrophic diagnosis, along with the findings of the ultrasound. The surgical team indicated emergency open cholecystectomy; balanced general anesthesia was used, with an estimated blood loss of 50 mL. Intraoperatively, approximately 100 mL of hemoperitoneum was found alongside a hematoma at the GB fundus with an adherent omental plastron. The GB was tensely distended, positioned intrahepatically, measuring 8 x 6 x 5 cm with 5-mm walls (Figure 1).

**Figure 2:**
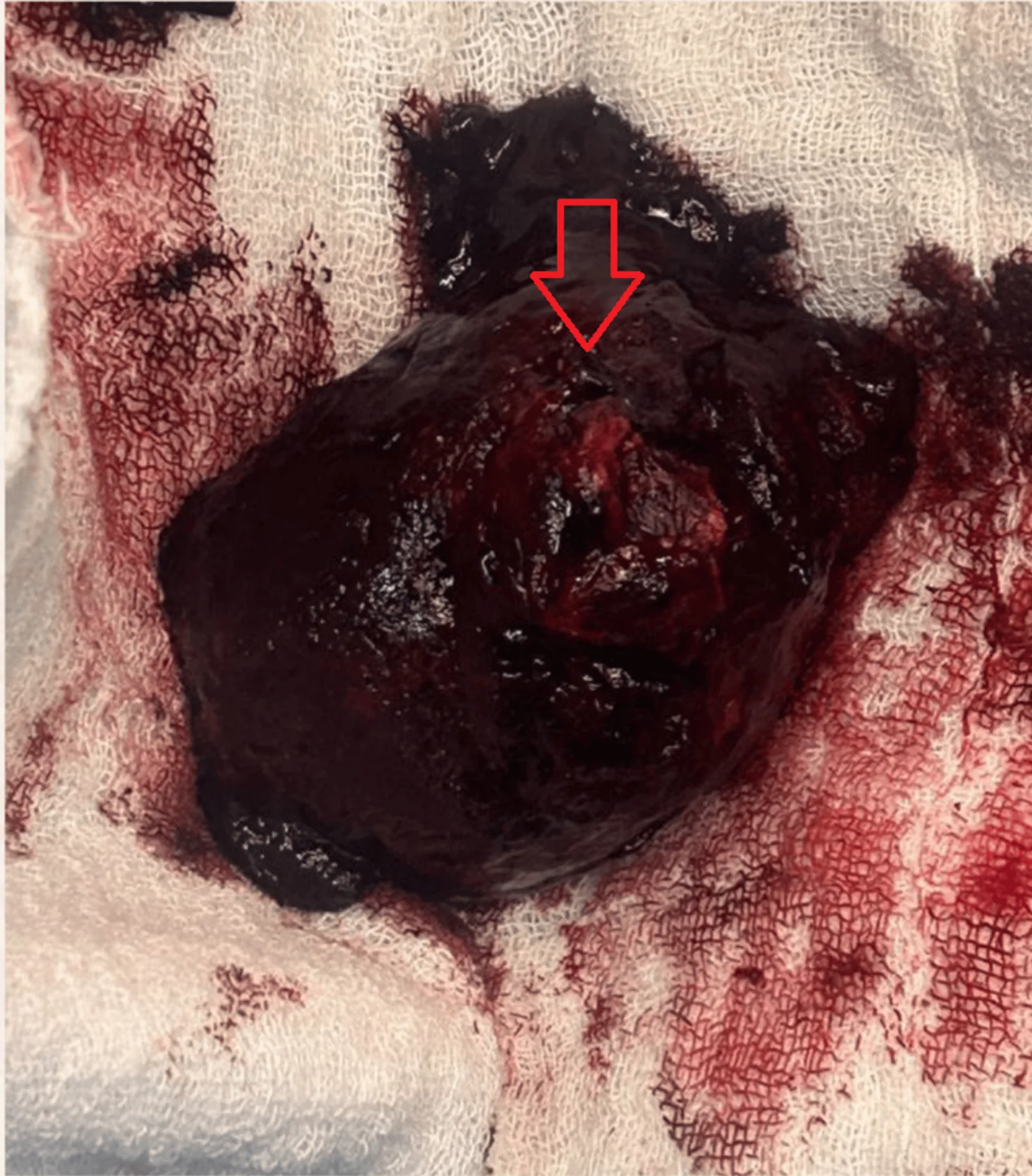
An organized clot (arrowhead) extracted from the gallbladder lumen during emergency cholecystectomy. The clot structure is consistent with subacute hemorrhage.

**Figure 3:**
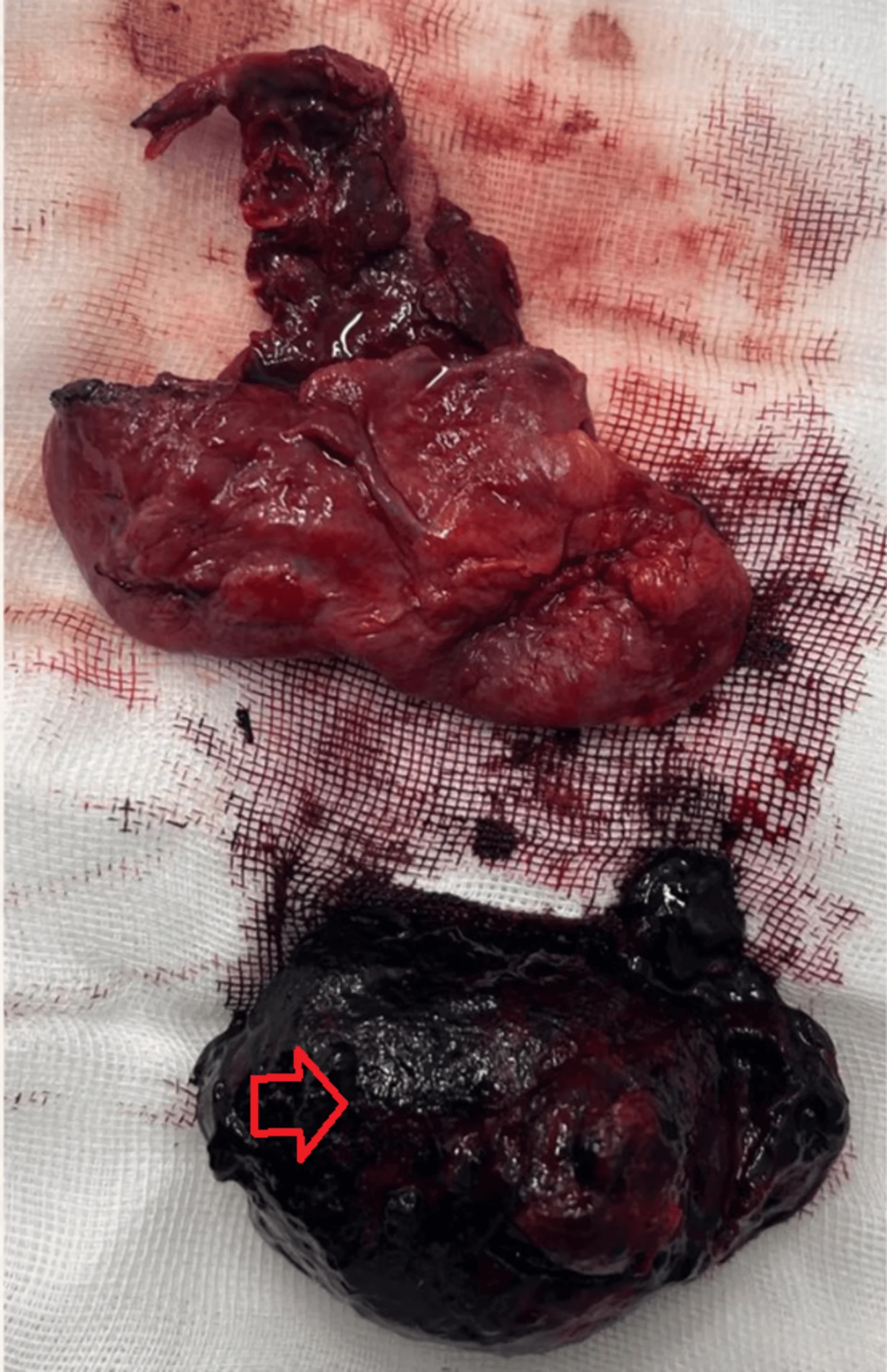
The excised gallbladder shows hemorrhagic content and an organized intraluminal clot (arrowhead). No cholelithiasis was identified, confirming acalculous etiology.

An organized intraluminal clot was identified and extracted (Figures 2, 3). No gallstones were found, confirming acalculous etiology.

The patient's postoperative recovery was uneventful. He required no vasopressor support and maintained stable hemodynamics throughout. He was discharged on postoperative day 2 with clinically satisfactory improvement. On the subsequent follow-up, two weeks later, the patient was still seen moving with the help of crutches but with companions, presenting satisfactory improvement, and therefore it was decided to discharge him from the general surgery service.

## Discussion

Hemorrhagic cholecystitis can progress within hours toward GB perforation, hemoperitoneum, and hypovolemic shock. Bleeding into the GB can manifest as perforation with or without hemoperitoneum, hemobilia with or without obstruction of the biliary tract, and gastrointestinal bleeding as hematemesis and melena [2,3]. This spectrum of possible presentations is part of what makes this condition easy to miss: the clinical picture changes depending on which complication predominates. Reported risk factors associated with hemorrhagic cholecystitis include anticoagulation therapy, malignancies, coagulopathies, history of trauma, renal failure, and cirrhosis. Khan Hotak et al. reported that among 6,458 patients who underwent cholecystectomy between 2000 and 2021, only 35 were diagnosed with hemorrhagic cholecystitis, yielding an overall incidence of 0.55%, with 88.6% presenting as emergency cases [5]. Published series document cases of the calculous form; though Huffman and Schenker describe multiple pathophysiologies and etiologies of acalculous cases, no indexed report has previously described acalculous hemorrhagic cholecystitis arising in the context of geriatric fall syndrome [4].

The mechanism in this patient is traceable. The right hemithorax and flank absorbed direct impact during the fall, and we consider this blunt trauma the most plausible precipitant of intraluminal hemorrhage. On rare occasions, hemorrhagic cholecystitis has been reported with acalculous cholecystitis, and trauma is among the documented precipitants; the specific association with fall syndrome and fall-related direct abdominal impact, however, has not been characterized. The patient's DFRI score placed him firmly within the geriatric fall syndrome category [8]. The Downton scale assesses fall risk across five domains; scores above two points indicate high risk, with a sensitivity of 0.58 and specificity of 0.62 in hospitalized patients [8]. Our patient scored three or more, reflecting three of the five domains simultaneously: unsafe gait due to his ankle fracture and crutch dependence, a previous fall requiring hospital admission, and sensory impairment related to his epilepsy. A longitudinal observational study by Downton and Andrews demonstrated that a DFRI cutoff of three or more points was significantly associated with fall-related injury after discharge, with an incidence rate ratio of 1.94 (95% confidence interval (CI): 1.60 to 2.38); among individual modules, previous falls and unsafe gait carried the strongest predictive weight [8]. While also described by Bueno-Garcia et al, this profile, when combined with blunt abdominal impact from a fall, created the clinical scenario we described [7].

The differential diagnosis in this case extended beyond the biliary tract and required systematic exclusion of competing diagnoses before the surgical team could commit to cholecystectomy as the definitive intervention. Hepatic subcapsular hematoma, splenic laceration, retroperitoneal hemorrhage, and traumatic hemobilia were all plausible given the mechanism of right-flank impact during a fall, and each has a distinct management process. The distinguishing feature on abdominal ultrasound - a distended GB with hyperdense intraluminal content and no acoustic shadowing, alongside free peritoneal fluid - is what directed the clinical team toward a biliary source rather than parenchymal injury. This finding is consistent with the sonographic pattern described by Gallaher and Charles and Huffman and Schenker, and subsequently referenced in case series of hemorrhagic cholecystitis, where the absence of acoustic shadowing differentiates intraluminal blood from gallstones and represents the most actionable imaging criterion in the emergency setting [3,4]. The decision to proceed with open rather than laparoscopic cholecystectomy reflects the degree of hemoperitoneum and the institutional context: in a resource-limited public hospital managing an 81-year-old with hemodynamic compromise, open access offered faster source control with lower risk of conversion-related delay, a rationale supported by current guidelines on emergency cholecystectomy in high-risk patients [9,10]. Comparing this case with the nearest published precedent - Gallaher's and Charles's report of acalculous hemorrhagic cholecystitis in an elderly anticoagulated male managed laparoscopically - the two cases share the acalculous etiology and the absence of cholelithiasis on histology, but differ in the precipitating mechanism (anticoagulation versus trauma), the degree of hemoperitoneum, and the surgical approach chosen [3]. No previous report identified in our literature search documents this condition arising from a ground-level fall in a patient with a validated high fall-risk score, which is the specific clinical context that this case contributes to the indexed literature.

The acalculous etiology was confirmed intraoperatively by the absence of gallstones. Hemorrhagic cholecystitis is estimated to affect between 0.55% and 7% of cholecystitis cases, with identified risk factors including underlying malignancy, anticoagulant therapy, and history of trauma; our patient's case fits the traumatic mechanism without the more commonly cited pharmacological or oncological cofactors [3,4]. Emergency open cholecystectomy resolved the hemorrhagic source, evacuated the hemoperitoneum, and led to discharge on postoperative day 2 without vasopressor support. Acute hemorrhagic cholecystitis with GB rupture can produce massive intra-abdominal hemorrhage, making early surgical intervention the most direct determinant of survival. This result, achieved in an 81-year-old with multiple comorbidities at a resource-limited public hospital, reflects what timely decision-making can accomplish in this population [9,10].

The DFRI deserves specific mention as a tool with implications beyond inpatient fall prevention. The DFRI consists of five modules: previous falls, medication, sensory deficits, mental state, and gait; three or more points indicate an increased fall risk. In a retrospective analysis of 469 patients who fell during a five-year period at a public hospital, the Downton scale showed an odds ratio of 2.31 for predicting falls, confirming its utility as a stratification tool in acute care settings [8]. What the present case adds is a surgical dimension to that risk: a patient who falls repeatedly and sustains direct abdominal impact is not only at risk of fractures or head injury - he is also at risk of visceral complications that may present days later, masked by concurrent neurological or traumatic diagnoses. The data on the incidence of falls and the profiles of patients who fell confirm that the Downton scale captures a clinically meaningful phenotype, and that phenotype should now be considered a context in which hemorrhagic biliary disease enters the differential [6-8].

As populations continue to age, fall syndrome is expected to become more prevalent. Perforated acalculous hemorrhagic cholecystitis has been described in settings of systemic vulnerability, such as renal failure and dialysis, suggesting that any condition that compromises normal physiological response to injury, whether metabolic or traumatic, can precipitate this complication [5]. Right-flank or right hemithorax trauma from a fall should now be included in the list of clinical scenarios where hemorrhagic cholecystitis enters the differential, particularly when unexplained right upper quadrant pain develops days after the initial trauma. Prospective registries documenting fall-related abdominal complications in elderly patients with high DFRI scores are needed to better characterize this association [11,12].

Regarding follow-up, the patient was discharged on postoperative day 2 with stable hemodynamics and no immediate complications. Outpatient review confirmed clinical recovery, though long-term hepatobiliary imaging was not performed given the confirmed acalculous etiology and the absence of residual biliary symptoms at the time of this report.

## Conclusions

This case demonstrates that hemorrhagic cholecystitis can present as a delayed complication of blunt right-flank trauma, emerging five days after a ground-level fall rather than immediately. In geriatric patients with a high DFRI who develop unexplained right upper quadrant pain after a fall with flank impact, acalculous hemorrhagic cholecystitis should remain in the differential even when initial imaging is inconclusive. Abdominal ultrasound alone was sufficient to guide emergency surgical decision-making in this hemodynamically borderline patient, without the need for computed tomography confirmation. Early recognition and prompt cholecystectomy achieved a favorable outcome despite the patient's advanced age and comorbidity burden.
